# Metabolite identification of salvianolic acid A in rat using post collision-induced dissociation energy-resolved mass spectrometry

**DOI:** 10.1186/s13020-024-00931-z

**Published:** 2024-04-26

**Authors:** Han Li, Ke Zhang, Wei Chen, Yuxuan Zhou, Jun Li, Yunfang Zhao, Yuelin Song

**Affiliations:** 1 Modern Research Center for Traditional Chinese Medicine, Beijing Research Institute of Chinese Medicine, Beijing University of Chinese Medicine, Beijing, 102488 China; 2 School of Chinese Materia Medica, Beijing University of Chinese Medicine, Beijing, 102488 China

**Keywords:** Salvianolic acid A, Metabolites, Ester bond dissociation, Post collision-induced dissociation energy-resolved mass spectrometry

## Abstract

**Background:**

As one of the most famous natural products, salvianolic acid A (SAA) is undergoing clinical trials for the treatments of angina pectoris and coronary heart disorders. However, the in vivo metabolites of SAA have only been tentatively identified, leading to a barrier for precise therapeutical drug monitoring.

**Methods:**

Ultra-high performance liquid chromatography coupled with quadrupole time of flight tandem mass spectrometry (UPLC–Qtof-MS/MS) was firstly employed to acquire high-resolution MS^1^ and MS^2^ spectra for all metabolites. Through paying special attention onto the features of ester bond dissociation, metabolism sites were restricted at certain regions. To further determine the metabolism site, such as the monomethylated products (**M23**, **M25**, and **M26**), post collision-induced dissociation energy-resolved mass spectrometry (post-CID ER-MS) was proposed through programming progressive exciting energies to the second collision chamber of hybrid triple quadrupole-linear ion trap mass spectrometry (Qtrap-MS) device.

**Results:**

After SAA oral administration, 29 metabolites (**M1**–**M29**), including five, thirteen, and sixteen ones in rat plasma, urine, and feces, respectively, were detected in rats. The metabolism route was initially determined by applying well-defined mass fragmentation pathways to those HR-*m*/*z* values of precursor and fragment ions. Metabolism site was limited to SAF- or DSS-unit based on the fragmentation patterns of ester functional group. Through matching the dissociation trajectories of concerned 1^st^-generation fragment ions with expected decomposition product anions using post-CID ER-MS strategy, **M23** and **M25** were unequivocally assigned as 3'-methyl-SAA and 3''-methyl-SAA, and **M26** was identified as 2-methyl-SAA or 3-methyl-SAA. Hydrolysis, methylation, glucuronidation, sulfation, and oxidation were the primary metabolism channels being responsible for the metabolites' generation.

**Conclusion:**

Together, the metabolism regions and sites of SAA metabolites were sequentially identified based on the ester bond dissociation features and post-CID ER-MS strategy. Importantly, the present study provided a promising way to elevate the structural identification confidence of natural products and metabolites.

**Graphical abstract:**

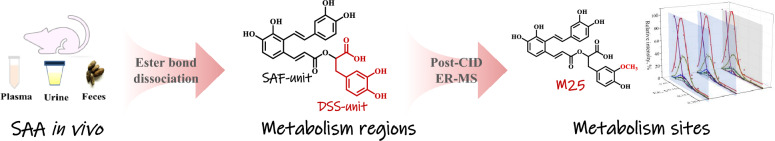

**Supplementary Information:**

The online version contains supplementary material available at 10.1186/s13020-024-00931-z.

## Background

Salvianolic acid A (SAA), sourced from Miltiorrhizae Radix *et.* Rhizoma (Chinese name: Danshen), is reputed as one of the most famous natural products [1]. As the primary contributor for the therapeutic benefit of Danshen, SAA exhibits a broad pharmacological spectrum, including anti-tumor effects [2, 3], myocardial protection [4, 5], hepatoprotective activity [6, 7], and modulation of gut microbiota imbalance [8]. It is worth noting that a new drug candidate derived from SAA is currently undergoing phase II clinical trials for the treatments of angina pectoris and coronary heart disorders. Because of the great absorption property and nonetheless, low oral bioavailability, the metabolites of SAA are believed to be the active forms in vivo and responsible for the therapeutic outcomes *via* interactions with drug targets [9, 10]. To our knowledge, the metabolism of SAA in vivo remains insufficiently investigated in comparison to its well-defined pharmacological effects. Therefore, in-depth clarification of SAA metabolism is a prerequisite to disclose the underlying therapeutic mechanisms and facilitate the development of potentially active drug candidates from metabolites.

**Fig. 1:**
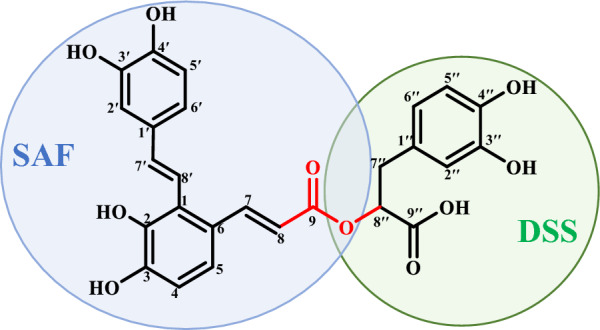
The chemical structure of salvianolic acid A (SAA)

SAA (Fig. 1) contains a total of six hydroxy moieties and one carboxyl moiety, all of which serve as the metabolism labile sites [11–13]. Notably, various metabolism sites lead to the widespread presence of isomers, resulting in that the confirmative annotation is crucial and challenging. Using nuclear magnetic resonance (NMR), confirmative identification of isomers can be achieved [9], but the application is constrained by limited sensitivity and labor- and time-consuming shortcomings, as well as risks of metabolite lability. LC–HR-MS/MS enables the determination of metabolic type by applying mass fragmentation pathways to those HR-*m*/*z* values obtained from MS^1^ and MS^2^ spectra [14–16]. However, definitive identification of SAA metabolites is difficult to achieve, because the isomers usually share identical MS^1^ and indistinguishable MS^2^ spectra. For example, methylated SAA were ambiguously assigned without specifying the substitution site [17, 18]. Online energy-resolved (ER)-MS [19, 20] and full collision energy ramp-MS^2^ (FCER-MS^2^) spectrum [21–25] have been proposed to investigate the differential MS/MS behaviors among isomers. In addition to the *m*/*z* values of precursor and fragment ions, the FCER-MS^2^ spectrum contains orthogonal information, such as the collision energy at 50% survival yield (CE_50_) of the precursor ion, optimal collision energy (OCE), and maximum relative ion intensity at OCE (RII_max_) of each fragment ion. Combined with quantum chemical calculations, OCE and RII_max_ of identical ion transitions for isomers are positively and negatively correlated with bond dissociation energy (BDE) and thermal enthalpy, respectively. Isomeric differentiation relies on aligning the ranking patterns of OCEs and RII_max_ of detected compounds with the corresponding BDEs and thermal enthalpy values of the candidate structures. Unfortunately, not all candidate structures can be detected simultaneously in practical samples, especially when extensive substitution sites exist, which means that FCER-MS^2^ strategy still has certain limitations in confirmatively identifying isomeric structures. Therefore, it is necessary to develop novel strategies for acquiring auxiliary structural information and advancing the identification of SAA metabolites in vivo.

The difficulty of structural identification can be reduced by hydrolyzing conjugated compound into decomposition products (*i.e.*, biosynthetic pioneers) and then comparing them with appropriate authentic simple compounds [26, 27]. We aimed to implement a similar concept in MS with improved sensitivity, specificity, and throughput [28]. The hybrid triple quadrupole-linear ion trap mass spectrometry (Qtrap-MS) is equipped with two tandem-in-space collision cells, *i.e.*, q2 collision cell and linear ion trap (LIT) chamber, and the latter one allows for MS^3^ spectra acquisition. Inspired by that FCER-MS^2^ spectrum contains trajectories from precursor ion to 1^st^-generation fragment ions, we proposed post collision-induced dissociation energy-resolved MS (post-CID ER-MS) to obtain trajectories from selected 1^st^-generation fragment ion to 2^nd^-generation fragment ions [29, 30]. Following the post-CID ER-MS strategy, intensities of residual 1^st^-generation fragment ion and all its 2^nd^-generation fragment ions were collected at different exciting energies (EEs). After normalization and curve fitting, full exciting energy ramp-MS^3^ (FEER-MS^3^) spectrum of selected 1^st^-generation fragment ion was obtained, which included exciting energy at 50% survival yield (EE_50_) of 1^st^-generation fragment ion, optimal exciting energy (OEE), and maximum relative ion intensity (RII_OEE_) of each 2^nd^-generation fragment ion [31, 32]. Theoretically, FEER-MS^3^ spectrum of 1^st^-generation fragment ion for conjugated structure should be same as FEER-MS^2^ spectrum of the (de)protonated ion for decomposition product bearing the expectational geometry, and *vice versa*.

SAA is the esterification product of salvianolic acid F (SAF) and danshensu (DSS), and similarly, hydrolysis of ester bond can yield the two biosynthetic pioneers, SAF and DSS (Fig. 1). Fortunately, the ester bond of SAA is also susceptible to cleavage in collision-induced dissociation (CID), resulting in a series of related fragment ions, including* a*^−^,* b*^−^, *c*^−^, *x*^−^, *y*^−^, and *z*^−^ ions according to the nomenclature rule [22]. Therefore, based on the ester bond dissociation features and post-CID ER-MS strategy, we proposed an analytical pipeline aiming to enhance the structural identification confidence and clarify the metabolic profile of SAA, following several progressive steps: (1) capturing HR-MS/MS information of SAA to analyze the features of ester bond dissociation; (2) obtaining HR-MS/MS information of SAA metabolites to determine the metabolism route and region (SAF- or DSS-unit) based on the mass fragmentation pathways and the features of ester bond dissociation, respectively; (3) acquiring FEER-MS^3^ spectra of targeted 1^st^-generation fragment ions for monomethylated SAA isomers using post-CID ER-MS strategy and comparing them with FEER-MS^2^ spectra of monomethylated SAF/DSS anions, to confirmatively identify the structures of monomethylated metabolites; and (4) proposing the possible metabolism channels for SAA. The obtained findings are expected to acquire a comprehensive metabolism map of SAA, and also demonstrate the significance of post-CID ER-MS strategy for elevating the structural annotation confidence.

## Materials and methods

### Chemicals and reagents

SAA, sodium DSS, and SAF were commercially supplied by Standard Technology Co., Ltd. (Shanghai, China). The purity of each compound was determined to be greater than 98% using LC–UV–IT-TOF–MS (Shimadzu, Kyoto, Japan). LC–MS grade formic acid, methanol, and acetonitrile (ACN) were purchased from Thermo-Fisher (Pittsburgh, PA). Deionized water was prepared in-house on a Milli-Q water purification instrument (Millipore, Bedford, MA). The other chemicals and reagents were of analytical grade and purchased from Beijing Chemical Co., Ltd. (Beijing, China).

### Sample collection and preparation

The animal study protocols were approved by the Animal Ethics Committee of Beijing University of Chinese Medicine (Beijing, China). Twenty-four male Sprague–Dawley rats (220 ± 20 g) were provided by Vital River Laboratory Animal Technology Co., Ltd. (Beijing, China). All animals were acclimated in an animal breeding room for 3 days with the temperature of 22 ± 2 °C, a relative humidity of 60 ± 5% and a 12 h light/dark cycle. Standard chow and Milli-Q water were provided ad libitum. All animals were fasted overnight and given free access to tap water prior to treatment. All rats were randomly divided into four groups (*n* = 6) that orally administrated (100 mg/kg for each compound) with SAA, sodium DSS, SAF, and vehicle (tap water), accordingly. The animals were individually housed in metabolic cages.

A 200 μL aliquot of blood was sampled from the orbital venous plexus of each rat at 0.5, 1, 2, 6, 12, and 24 h after oral administration. Urine and feces were collected individually from metabolic cages over 0–24 h in an ice water bath. Drug-free biological samples were collected from vehicle group in parallel. Plasma and urine were centrifuged under 3 000 rpm and 12 000 rpm, respectively, for 10 min at 4 °C, and supernatants were retained. Feces underwent lyophilization and grinding, successively. Thereafter, all samples were stored at −80 °C until usage.

After being thawed in ice-water bath, each sample type was pooled within each group to generate a single homogenized sample. Three volumes of ACN were fortified into 300 μL homogenized plasma or urine samples for protein precipitation and desalting. Ten volumes of methanol were utilized to extract pulverized feces with an ultrasonic-assisted manner. The supernatants of plasma samples were evaporated to dryness at 30 °C under gentle nitrogen stream, and the residues were reconstituted with 50 μL of 25% aqueous ACN, vortexed for 2 min, and centrifugated at 12 000 rpm for 15 min at 4 °C, successively. Following thorough vortex and centrifugation, the supernatants of urine and feces were individually filtered with 0.22 μm nylon membranes.

### LC–MS/MS measurements

All biological samples participated in LC–MS/MS measurements. All chromatographic separations were conducted on Shimadzu LC modular system (Kyoto, Japan) equipped with a Waters ACQUITY UPLC HSS T3 column (2.1 × 100 mm, 1.8 μm, Milford, CT). Mobile phase consisting of 0.1% aqueous formic acid (A) and ACN (B) was delivered in gradient at a total flow rate of 0.2 mL/min by operating the program as follows: 0–4 min, 2–10% B; 4–20 min, 10–30% B; 20–25 min, 30–65% B; 25–28 min, 65–100% B; and 28–32 min, 100% B. After each analytical run, 2% B was delivered for another five minutes for the re-equilibration. Column oven and injection volume were set as 40 °C and 2 μL, respectively.

The column outlet was directly connected to ESI interface of either Qtof-MS (TripleTOF 6600^+^, SCIEX, Foster City, CA) or Qtrap-MS (5500 Qtrap-MS, SCIEX) device. Actually, the whole measurement consisted of three progressive steps: UPLC–Qtof-MS/MS for acquiring HR-*m*/*z* values of precursor and fragment ions, online ER-MS for obtaining OCEs of the targeted 1^st^-generation fragment ions, and post-CID ER-MS for gaining the trajectories from selected 1^st^-generation fragment ions to 2^nd^-generation fragment ions.

#### Qtof-MS assays

The ion source parameters were set as follows: ion-spray needle voltage (IS), −4500 V; nebulizer (GS1), heater (GS2), and curtain gas flow rate, 55, 55, and 30 units, respectively; heater gas temperature (TEM), 500 °C. Mass range as 100–1000 Da was defined to record MS^1^ and MS^2^ spectra. MS^2^ spectra were acquired by data-dependent acquisition (DDA) manner with CE as −30 eV, collision energy spread (CES) as 15 eV. SCIEX PeakView1.2 software was utilized for data processing.

#### Qtrap-MS assays

The online ER-MS and post-CID ER-MS were configured using MRM and MS^3^ scan modes of Qtrap-MS. A methylation product namely **M25** was taken as a case to clarify the detailed settings. Based on the fragmentation patterns of ester functional group in CID [33], the fragment ions at *m*/*z* 313.07, 295.06, 211.06, and 193.05 were assigned as *c*^−^, *b*^−^,* y*^−^, and *z*^−^, respectively, in MS^2^ spectrum of *m*/*z* 507.13 ([M–H]^−^) (Table 1). The *c*^−^ and *b*^−^ ions had identical *m*/*z* values with [M_SAF_–H]^−^ and [M_SAF_–H–H_2_O]^−^, while *y*^−^ and *z*^−^ ions showed an increase of 14 Da (CH_2_ fortification) compared to [M_DSS_–H]^−^ and [M_DSS_–H–H_2_O]^−^. Hence, it could be deduced that the methylation site was located at DSS-unit. Because our concern was the comparability between certain fragment ions and the deprotonated ions of those decomposition products, subsequent experiments focused only on the *m*/*z* 313.0676 (*c*^−^) and 211.0638 (*y*^−^) ions. The data recording and processing were performed using SCIEX Analyst software (Version 1.6.3).

**Table 1:** UPLC–Qtof-MS/MS chromatographic and spectrometric information of in vivo metabolites (**M1**–**M29**) of SAA

No.	*t*_R_ (min)	Formula	[M–H]^−^	Error (ppm)	Fragments ions with characteristic ions	Identity	Distribution*
SAA	23.89	C_26_H_22_O_10_	493.1145	1.0	**313.0736 (*c***^−^**)**, **295.0619(*b***^−^**)**, **269.0812(*a***^−^**)**, 203.0350, **197.0472(*y***^−^**)**, 185.0249, **179.0348(*z***^−^**)**, 159.0458, 135.0444, 109.0303	salvianolic acid A (SAA)	U, F
M1	7.67	C_9_H_10_O_5_	197.0457	0.8	135.0431, 123.0458, 72.9924	danshensu (DSS)	F
M2	18.15	C_23_H_22_O_12_	489.1050	2.4	313.0730, 269.0854, 159.0466, 109.0292	SAF glucuronide	U
M3	19.73	C_17_H_16_O_6_	315.0873	−0.4	271.0988, 255.0616, 135.0448, 109.0238	hydrogenated SAF	U
M4	20.15	C_17_H_14_O_6_	313.0725	1.4	269.0759, 251.0773, 159.0423, 135.0474, 109.0295	salvianolic acid F (SAF)	U
M5	20.54	C_32_H_30_O_16_	669.1462	0.1	493.1144, **471.1015(*b***^−^**)**, 313.0606, 295.0626, 185.0229, 109.0315	[SAF-glucuronide]-[DSS]	U, F
M6	20.91	C_32_H_28_O_16_	667.1315	1.6	491.0973, **487.0981(*c***^−^**)**, 311.0582, 293.0505, 135.0507	[tournefolic acid A- glucuronide]-[DSS]	F
M7	21.52	C_32_H_30_O_16_	669.1481	3.0	493.1222, **471.0898(*b***^−^**)**, **445.0998(*a***^−^**)**, 313.0785, 295.0647, 185.0251	[SAF-glucuronide]-[DSS]	U, F
M8	21.94	C_32_H_30_O_16_	669.1462	0.1	493.1160, **489.0967(*c***^−^**)**, **445.1225(*a***^−^**)**, 295.0651, 185.0241, 109.0272	[SAF-glucuronide]-[DSS]	F
M9	22.42	C_32_H_28_O_16_	667.1291	−2.0	491.0988, **355.0668(*y***^**−**^**)**, 311.0474, 293.0488, 267.0780, 197.0450, 179.0405, 175.0255, 135.0477	[tournefolic acid A]-[DSS-glucuronide]	F
M10	22.55	C_26_H_22_O_13_S	573.0691	−3.0	493.1074, **393.0397(*c***^−^**)**, 295.0658, **197.0425(*y***^−^**)**, 185.0251, 109.0286	[SAF-sulfate]-[DSS]	F
M11	22.75	C_33_H_32_O_16_	683.1618	0.1	507.1259, **471.0983(*b***^−^**)**, 313.0721, 295.0665, 185.0255, 109.0267	[methyl-DSS]-[SAF-glucuronide]	U
M12	23.14	C_26_H_22_O_13_S	573.0719	1.9	493.1062, 295.0644, 185.0236, 135.0447	SAA sulfate	U
M13	23.32	C_26_H_22_O_13_S	573.0692	−2.9	493.0975, **393.0393(*c***^−^**)**, 295.0595, 185.0231, 159.0429, 109.0291	[SAF-sulfate]-[DSS]	F
M14	23.68	C_33_H_32_O_16_	683.1620	0.4	507.1001, **471.1004(*b***^−^**)**, 295.0581, 185.0286	[methyl-DSS]-[SAF-glucuronide]	U
M15	24.06	C_32_H_28_O_16_	667.1281	−3.5	491.1116, **469.0669(*b***^−^**)**, 311.0563, 293.0453, **197.0513(*z***^**−**^**)**, 135.0404	[tournefolic acid A- glucuronide]-[DSS]	F
M16	24.66	C_34_H_34_O_16_	697.1747	−3.9	521.1539, 485.1085, **327.0944 (*c***^**−**^**–GluA or *c***^**−**^**)**, 309.0765, **211.0746(*y***^**−**^** or *y***^**−**^**–GluA)**	[methyl-SAF]-[methyl-DSS]-glucuronide	U
M17	24.68	C_34_H_34_O_16_	697.1780	0.8	521.1510, **327.1000(*c***^**−**^**–GluA or *c***^**−**^**)**, 309.0794, **211.0610(*y***^**−**^** or *y***^**−**^**–GluA)**	[methyl-SAF]-[methyl-DSS]- glucuronide	P
M18	25.07	C_34_H_34_O_16_	697.1776	0.3	521.1494, 503.117, **327.0911(*c***^**−**^**–GluA or *c***^**−**^**)**, 309.0768, 294.0526, 278.0613, **211.0630(*y***^**−**^** or *y***^**−**^**–GluA)**, **193.0510(*z***^**−**^** or* z***^**−**^**–GluA)**	[methyl-SAF]-[methyl-DSS]- glucuronide	P
M19	25.32	C_26_H_20_O_10_	491.0990	1.3	**311.0573(*c***^−^**)**, **293.0467(*b***^−^**)**, **267.0669(*a***^−^**)**, 265.0510, **197.0480(*y***^−^**)**, **179.0365(*z***^−^**)**, 135.0456	salvianolic acid C (SAC)	F
M20	25.42	C_34_H_34_O_16_	697.1776	0.3	521.1506, **327.0913(*c***^**−**^**–GluA or *c***^**−**^**)**, **309.0755(*b***^**−**^**–GluA or *b***^**−**^**)**, 294.0585, **211.0613(*y***^**−**^** or *y***^**−**^**–GluA)**	[methyl-SAF]-[methyl-DSS]- glucuronide	P
M21	25.52	C_26_H_22_O_9_	477.1175	−3.4	**313.0717(*c***^−^**)**, **295.0591(*b***^−^**)**, 185.0258, 159.0484, 109.0289	[SAF]-[DSS-dehydroxylated]	F
M22	25.54	C_34_H_34_O_16_	697.1792	2.6	521.1270, **309.0749(*b***^**−**^**–GluA or *b***^**−**^**)**, 283.0912	di-methyl-SAA glucuronide	P
M23	25.61	C_27_H_24_O_10_	507.1291	−1.1	**327.0923(*c***^−^**)**, **309.0728(*b***^−^**)**, **197.0456(*y***^−^**)**, **179.0336(*z***^−^**)**, 135.0468	3'-methyl-SAA	F
M24	25.70	C_34_H_34_O_16_	697.1783	1.3	521.1426, **327.0860(*c***^**−**^**–GluA or *c***^**−**^**)**, **309.0749(*b***^**−**^**–GluA or *b***^**−**^**)**, 294.0522, 283.1019, **211.0590(*y***^**−**^** or *y***^**−**^**–GluA)**	[methyl-SAF]-[methyl-DSS]- glucuronide	P
M25	25.78	C_27_H_24_O_10_	507.1300	0.6	**313.0676(*c***^−^**)**, **295.0631(*b***^−^**)**, **211.0638(*y***^−^**)**, 203.0346, **193.0523(*z***^−^**)**, 185.0227, 109.0273	3''-methyl-SAA	U
M26	25.96	C_27_H_24_O_10_	507.1293	−0.7	**327.0863(*c***^−^**)**, **309.0833(*b***^−^**)**, 294.0554, 278.0731, **197.0471(*y***^−^**)**, **179.0333(*z***^−^**)**	2-methyl-SAA or 3-methyl-SAA	U, F
M27	26.42	C_26_H_20_O_9_	475.1032	−0.5	**311.0710(*c***^−^**)**, **293.0407(*b***^−^**)**, 119.0511	[tournefolic acid A]-[DSS-dehydroxylated]	F
M28	26.73	C_28_H_26_O_10_	521.1449	−0.8	**327.0934(*c***^−^**)**, **309.0776(*b***^−^**)**, 294.0480, 277.0507, **211.0644(*y***^−^**)**, **193.0489(*z***^−^**)**	[methyl-SAF]-[methyl-DSS]	U, F
M29	26.89	C_28_H_26_O_10_	521.1435	−3.5	**327.0866(*c***^−^**)**, **309.0782(*b***^−^**)**, 294.0600, 277.0490, **211.0608(*y***^−^**)**, **193.0545(*z***^−^**)**, 165.0535	[methyl-SAF]-[methyl-DSS]	U, F

^*^P plasma,* U* urine,* F* feces; characteristic ions were indicated in bold

The previously described online ER-MS [21] was strictly followed to gain OCEs for the concerned MS^2^ fragment ions (*c*^−^ and *y*^−^ ions). For the ion transition *m*/*z* 507.13 > 211.06, a set of pseudo-ion transitions (PITs) was derived, such as *m*/*z* 507.100 > 211.100, 507.100 > 211.101, 507.100 > 211.102, *etc*., corresponding to progressive CEs ranging from −5 to −81 eV. The responses under different CEs were imported into GraphPad Prism 8.0 software (San Diego, CA) to plot Gaussian-shaped breakdown graphs. The obtained OCE was used for subsequent MS^3^ data acquisition.

The follow-up post-CID ER-MS strategy in MS^3^ mode involved two CID, which occurred sequentially in q2 and LIT, corresponding to energies called CE and EE, respectively. OCE was set in q2 to accumulate enough fragment ions for MS^3^ acquisition. To distinguish fragment ions generated in q2 and LIT, we referred to them as 1^st^-generation fragment ions and 2^nd^-generation fragment ions, respectively [34]. Precursor ion, concerned 1^st^-generation fragment ion, and 2^nd^-generation fragment ions of each compound were involved to construct Q1 > Q3 > Q_LIT_ ion transitions [29–31]. Further fragmentation trajectories of *m*/*z* 211.06 for **M25** were obtained at progressive absolute values of EE ranging from 0.05 to 0.30 V in LIT. The breakdown graphs of residual 1^st^-generation fragment ion (*m*/*z* 211.06) and 2^nd^-generation ions (*m*/*z* 211.06, 193.00, 178.00, 165.00, 150.00, 149.10, and 134.00) were fitted using sigmoid plot and multiple iterations with OriginPro9 software (OriginLab, Northampton, MA), respectively. Thereafter, breakdown graphs were assembled and normalized to obtain the FEER-MS^3^ spectrum of *m*/*z* 211.06 for **M25**, which implied molecular descriptors (MDs), including EE_50_, OEE, and RII_OEE_.

The related decomposition products, monomethylated DSS (**D14** and **D15**), which had the same *m*/*z* value as the *y*^−^ of monomethylated SAA (**M25**), were also involved in post-CID ER-MS measurements. The ion transition *m*/*z* 211.06 > 211.06 > Q_LIT_ of **D14** and **D15** was employed to produce breakdown graphs. Under such status, the MS^2^ spectrum generated through CID in the LIT cell and the resultant breakdown graph set was termed as FEER-MS^2^ spectrum [31].

## Results and discussion

### Mass fragmentation behaviors of SAA

The fragmentation behaviors of SAA were investigated by employing UPLC–Qtof-MS/MS. The deprotonated ion ([M–H]^−^) of SAA was observed at *m*/*z* 493.11, corresponding to the elemental composition as C_26_H_22_O_10_. After converting HR-*m*/*z* to elemental compositions, the fragment ions in MS^2^ spectrum were assigned as* m*/*z* 313.07 [M–H–C_9_H_8_O_4_]^−^, 295.06 [M–H–C_9_H_10_O_5_]^−^, 269.08 [M–H–C_10_H_8_O_6_]^−^, 203.04 [M–H–C_9_H_8_O_4_–C_6_H_6_O_2_]^−^, 197.05 [M–H–C_17_H_12_O_5_]^−^, 185.02 [M–H–C_9_H_10_O_5_–C_6_H_6_O_2_]^−^, 179.03 [M–H–C_17_H_14_O_6_]^−^, 159.05 [M–H–C_10_H_8_O_6_–C_6_H_6_O_2_]^−^, 135.04 [M–H–C_18_H_14_O_8_]^−^, and 109.03 [M–H–C_20_H_16_O_8_]^−^ (Additional file 1: Fig. S1A). Referring to the chemical structure of SAA, the dissociations around the ester bond were regarded as the primary contributions towards the entire mass fragmentation pattern. Similar to the nomenclature rule of peptides, a nomenclature rule was proposed for fragment ions related to the ester bond dissociation, including *a*^−^,* b*^−^,* c*^−^,* x*^−^,* y*^−^, and *z*^−^ [22]. Based on the mathematical relationships between complementary ions (such as *a*^−^ and *x*^−^, *b*^−^ and *y*^−^, as well as *c*^−^ and *z*^−^), along with the unique mass differences between *a*^−^ and *b*^−^ (CO, 25.98 Da), *b*^−^ and *c*^−^ (H_2_O, 18.01 Da) [33], the featured ions *m*/*z* 269.08, 295.06, 313.07, 197.05, and 179.03 were assigned as *a*^−^, *b*^−^, *c*^−^, *y*^−^, and *z*^−^, respectively. The fragmentation pathways of SAA are proposed in Additional file 1: Fig. S1B, which provide references for the follow-up identification of in vivo metabolites.

### Metabolite identification for SAA by applying ester bond dissociation pattern

**Fig. 2:**
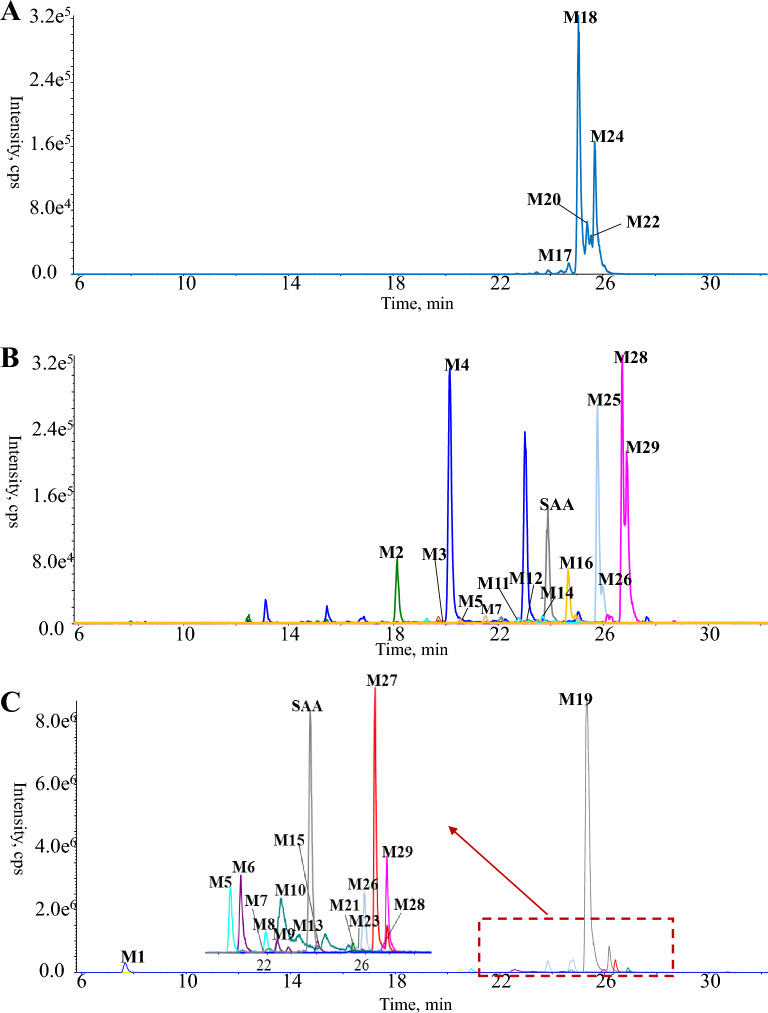
Overlaid extracted ion current chromatograms of SAA-treated plasma (**A**), urine (**B**), and feces (**C**)

After carefully comparing each homogenized biological sample with the respective drug-free sample, a total of 29 metabolites (**M1**–**M29**) were detected. Thereof, five ones (**M17**, **M18**, **M20**, **M22**, and **M24**), thirteen ones (**M2**–**M5**, **M7**, **M11**, **M12**, **M14**, **M16**, **M25**, **M26**, **M28**, and **M29**), and sixteen ones (**M1**, **M5**–**M10**, **M13**, **M15**, **M19**, **M21**, **M23**, and **M26**–**M29**) were distributed in plasma, urine, and feces, respectively. The detailed chromatographic and mass spectrometric information is summarized in Table 1, and representative chromatograms are illustrated in Fig. 2.

Among the metabolites, **M1**–**M4** were derived from hydrolysis of SAA and identified as DSS, SAF glucuronide, hydrogenated SAF, and SAF, respectively. **M1** and **M4** were identified by comparing with literature and reference substances [35, 36]. The **M3** anion (*t*_R_, 19.73 min) existed at *m*/*z* 315.09 (C_17_H_16_O_6_), which was 2 Da greater than SAF anion. Additionally, typical fragment ions were observed at *m*/*z* 271.10 [M–H–CO_2_]^−^, 255.06 [M–H–C_2_H_4_O_2_]^−^, 135.05 [M–H–C_9_H_8_O_4_]^−^, and 109.02 [M–H–C_11_H_10_O_4_]^−^; therefore, **M3** was finally identified as hydrogenated SAF. The deprotonated ion ([M–H]^−^) of **M2** (*t*_R_, 18.15 min) was observed at *m*/*z* 489.11, corresponding to a molecular formula as C_23_H_22_O_12_, which was 176 Da (C_6_H_8_O_6_) greater than SAF anion. The fragment ions observed after a neutral loss of 176 Da were consistent with those of SAF, confirming the identification of **M2** as a glucuronidation product of SAF.

All other metabolites (**M5**–**M29**) were direct derivatives of SAA, and the metabolism regions, either SAF-unit or DSS-unit, were judged based on MS^2^ spectral information and the ester bond dissociation pattern. The presence of substitution at SAF-unit was determined by calculating the mass difference between *c*^−^, *b*^−^, and *a*^−^ ions of metabolites and the corresponding ions of SAA, and similarly, the substitution site at DSS-unit was determined by the mass difference between *z*^−^ and *y*^−^ ions of metabolites and their relevant ions of SAA.

Deprotonated ions of **M5** (*t*_R_, 20.54 min), **M7** (*t*_R_, 21.52 min), and **M8** (*t*_R_, 21.94 min) were observed at *m*/*z* 669.15, suggesting the molecular formula as C_32_H_30_O_16_ for each. Compared to SAA (molecular formula: C_26_H_22_O_10_), glucuronidation should be responsible for GluA (C_6_H_8_O_6_, 176 Da) fortification. In addition, the signals at *m*/*z* 493.11 ([M–H–GluA]^−^) in MS^2^ spectra of **M5**, **M7**, and **M8** further consolidated that these were glucuronidation products of SAA. Subsequent efforts were towards the identification of metabolism regions based on the pattern of ester bond dissociation. The *b*^−^ (*m*/*z* 471.10) of **M5** and **M7** exhibited 176 Da greater than *b*^−^ (*m*/*z* 295.06) of SAA, while *c*^−^ (*m*/*z* 489.10) and *a*^−^ (*m*/*z* 445.10) ions of **M8** also showed a similar increase by 176 Da compared to *c*^−^ (*m*/*z* 313.07) and *a*^−^ (*m*/*z* 269.08) ions of SAA, indicating that glucuronidation occurred at the SAF-unit ([SAF-glucuronide]-[DSS]) for all three ones.

**M10** (*t*_R_, 22.55 min), **M12** (*t*_R_, 23.14 min), and **M13** (*t*_R_, 23.32 min) were all assigned as SAA sulfate (molecular formula: C_26_H_22_O_13_S) attributing to the signals at *m*/*z* 573.07 ([M–H]^−^) in MS^1^ spectra and *m*/*z* 493.11 ([M–H–SO_3_]^−^) in MS^2^ spectra. The *c*^−^ (*m*/*z* 393.04 [M–H–C_9_H_8_O_4_]^−^) of **M10** and **M13** showed 80 Da greater than *c*^−^ of SAA, indicating that sulfation sites might located at the SAF-unit ([SAF-sulfate]-[DSS]). Nevertheless, because the MS^2^ spectrum of **M12** contained few characteristic ions, the conjugation region was not confirmed in the current study.

Three isomers, **M23** (*t*_R_, 25.61 min), **M25** (*t*_R_, 25.78 min), and **M26** (*t*_R_, 25.96 min), were all annotated as the methylation products of SAA, because their deprotonated ions at *m*/*z* 507.13 ([M–H]^−^, C_27_H_24_O_10_) were 14 Da (CH_2_) greater than SAA anion. Thereof, the featured ions* c*^−^ (*m/z* 327.09 [M–H–C_9_H_8_O_4_]^−^) and *b*^−^ (*m/z* 309.07 [M–H–C_9_H_10_O_5_]^−^) of **M23** and **M26** exhibited a mass increase of 14 Da compared to *c*^−^ and *b*^−^ ions of SAA, respectively. Meanwhile, the featured ions *y*^−^ (*m/z* 197.05 [M–H–C_18_H_14_O_5_]^−^) and* z*^−^ (*m/z* 179.03 [M–H–C_18_H_16_O_6_]^−^) of **M23** and **M26** were consistent with those observed in SAA. The above demonstrated that methylation region was located at SAF-unit ([methyl-SAF]-[DSS]). In addition, *m*/*z* 294.06 ([M–H–C_9_H_10_O_5_–CH_3_·]^−·^) of **M26** was generated by methyl radical cleavage (CH_3_·) of *b*^−^, further confirming the deduction about methylation region. While *y*^−^ (*m/z* 211.06) and *z*^−^ (*m/z* 193.05) of **M25** were found to be 14 Da greater than their counterparts of SAA, this proved that its methylation site was at the DSS-unit ([SAF]-[methyl-DSS]).

**M11** (*t*_R_, 22.75 min) and **M14** (*t*_R_, 23.68 min) shared identical precursor ions at *m*/*z* 683.16, and the molecular formula was consequently calculated as C_33_H_32_O_16_, implying that either could be the product of methylation plus glucuronidation of SAA. The fragment ions at *m*/*z* 507.10 in MS^2^ spectra were generated by the natural loss of 176 Da. **M11** and **M14** possessed almost identical fragment ions to **M5** ([SAF-glucuronide]-[DSS]), including *m*/*z* 471.10 (*b*^−^), 313.07 (*c*^−^–176 Da), and 295.06 (*b*^−^–176 Da), suggesting that glucuronidation site of **M11** and **M14** was located at the SAF-unit. Subsequently, based on the complementarity of *b*^−^ and *y*^−^ ion, the mass of *y*^−^ was presumed to be *m*/*z* 211.06, which was consistent with [M–H]^−^ of monomethylated DSS, indicating that the methylation site was at the DSS-unit. Together, **M11** and **M14** were identified as [methyl-DSS]-[SAF-glucuronide].

In the case of a pair of isomers, **M28** (*t*_R_, 26.73 min) and **M29** (*t*_R_, 26.89 min), their empirical formulas were all suggested as C_28_H_26_O_10_ based on the deprotonated ions at *m*/*z* 521.14 ([M–H]^−^). Compared to SAA, di-methylation should be responsible for two CH_2_ (28 Da) fortification. The MS^2^ spectra of **M28** and **M29** exhibited an identical set of fragment ions, including *c*^−^ (*m*/*z* 327.09), *b*^−^ (*m*/*z* 309.08), *y*^−^ (*m*/*z* 211.06), and *z*^−^ (*m*/*z* 193.05). Thereof, *c*^−^ and *b*^−^ were identical with *c*^−^ and *b*^−^ of **M23** and **M26** ([methyl-SAF]-[DSS]), respectively; meanwhile, *y*^−^ and *z*^−^ corresponded to those of **M25** ([SAF]-[methyl-DSS]). Hence, two methylation sites separately occurred at either unit of SAA, and finally, **M28** and **M29** were termed as [methyl-SAF]-[methyl-DSS].

The deprotonated ions ([M–H]^–^) of **M16** (*t*_R_, 24.66 min), **M17** (*t*_R_, 24.68 min), **M18** (*t*_R_, 25.07 min), **M20** (*t*_R_, 25.42 min), **M22** (*t*_R_, 25.54 min), and **M24** (*t*_R_, 25.70 min) occurred at *m*/*z* 697.18 (molecular formula: C_34_H_34_O_16_), indicating that they were resulted from fortifying two CH_2_ together with a glucuronyl group to SAA. In MS^2^ spectra of **M16**–**M18**, **M20**, and **M24**, the product ions at *m*/*z* 327.09, 309.08, 294.06, 211.07, and 193.05 corresponded to the featured ions of the di-methylation metabolites of SAA described above. Therefore, two methylation sites separately located at SAF-unit and DSS-unit, *i*.*e*., [methyl-SAF]-[methyl-DSS]-glucuronide. However, in the MS^2^ spectrum of **M22**, there was not enough characteristic fragment ions to support the determination of the methylation sites; therefore, **M22** was tentatively identified as di-methyl-SAA glucuronide. Unfortunately, neutral loss of GluA was observed prior to ester bond dissociation in CID, resulting in the absence of featured fragment ions, and as a consequence, it was challenging to determine the glucuronidation site.

The deprotonated molecular ion ([M–H]^–^) of **M21** (*t*_R_, 25.52 min) was observed at* m*/*z* 477.12 (C_26_H_22_O_9_), which was 16 Da less than SAA. Other featured ions in MS^2^ spectrum, such as *c*^−^ (*m*/*z* 313.07) and *b*^−^ (*m*/*z* 295.06) were consistent with the fragment ions of the prototype. Therefore, **M21** was identified as the dehydroxylated derivative of SAA and dehydroxylation occurred on the DSS-unit ([SAF]-[DSS-dehydroxylated]).

**M19** (*t*_R_, 25.32 min) possessed a [M–H]^−^ signal as* m*/*z* 491.10 (molecular formula: C_26_H_20_O_10_), 2 Da less than SAA, indicating it might be dehydrogenation product of SAA. The metabolism site was determined to be at the SAF-unit by comparing the featured fragment ions of **M19** and SAA, including *c*^−^ (*m*/*z* 311.05 *vs*. 313.07), *b*^−^ (*m*/*z* 293.05 *vs*. 295.06), *a*^−^ (*m*/*z* 267.06 *vs*. 269.08),* y*^−^ (*m*/*z* 197.05 *vs*. 197.05), and *z*^−^ (*m*/*z* 179.04 *vs*. 179.04). Through referring the descriptions in the literature [17, 36, 37], **M19** was definitely determined as salvianolic acid C (SAC).

**M6** (*t*_R_, 20.91 min), **M9** (*t*_R_, 22.42 min), and **M15** (*t*_R_, 24.06 min) shared an identical [M–H]^−^ ion at* m*/*z* 667.13 (C_32_H_28_O_16_), which was 176 Da greater than that of SAC, and their generation should be attribute to the glucuronidation for SAC. Meanwhile, in MS^2^ spectra of **M6** and **M15**, the featured ions including* b*^*−*^ (*m*/*z* 469.07) and* c*^*−*^ (*m*/*z* 487.10), were 176 Da greater than the *b*^*−*^ (*m*/*z* 293.05) and *c*^*−*^ (*m*/*z* 311.05) of SAC, respectively, which reminded that the glucuronidation sites might locate at the SAF-unit ([tournefolic acid A-glucuronide]-[DSS]). Nevertheless, the MS^2^ spectrum of **M9** displayed a characteristic ion of *y*^−^ (*m*/*z* 355.07), indicating that the conjugation site on the DSS-unit ([tournefolic acid A]-[DSS-glucuronide]).

The [M–H]^−^ ion of **M27** was observed at *m*/*z* 475.10 with a retention time of 26.42 min, and the molecular formula thereafter came out as C_26_H_20_O_9_. **M27** was 16 Da less than SAC, and as a result, dehydroxylation should be responsible for the metabolic process. Moreover, fragment ions *c*^−^ (*m*/*z* 311.07) and *b*^−^ (*m*/*z* 293.04) were detected in the MS^2^ spectrum, corresponding to *c*^−^ and *b*^−^ of SAC, implying that the metabolism region occurred at the DSS-unit. Consequently, **M27** was tentatively identified as dehydroxylated SAC ([tournefolic acid A]-[DSS-dehydroxylated]).

### Isomeric identification of methylation metabolites

Through analyzing HR-MS/MS spectral information, the metabolites of SAA in vivo were tentatively annotated. Thereafter, based on the ester bond dissociation features, one step forward was achieved through placing the metabolism site(s) onto SAF-unit and/or DSS-unit. To further advance structural identification confidence, three isomeric metabolites of methylation, such as **M23**, **M25**, and **M26**, were employed as representatives to elaborate structural identification details. Noteworthily, to aid structural identification for SAA metabolites, the metabolites of SAF (**F1**–**F30**) and DSS (**D1**–**D15**) were first characterized carefully through matching with the information archived in the literature [38–40]. Attributing to the relatively simple structures, confirmative identification was usually achieved, and their identities together with key MS/MS spectral information were summarized in Additional file 1: Tables S1, S2.

According to the annotation of SAA methylation region mentioned above, **M23** and **M26** occurred at the SAF-unit, while **M25** took place at the DSS-unit, but the specific metabolism site could not be determined, *e.g.*, either the C-3-OH or C-4-OH of DSS. Based on the fact that the *c*^−^ and *y*^−^ ions produced by ester bond dissociation should show identical structures to those of the hydrolysis product anions, we thereby employed the post-CID ER-MS strategy. The strategy was performed to determine the structures of the *c*^−^ and *y*^−^ ions by comparing the FEER-MS^3^ spectra of *c*^−^ and *y*^−^ ions for esters with FEER-MS^2^ spectra of [M–H]^–^ for candidate hydrolysis products. Therefore, the methylation metabolites (**F28** and **F29**, as well as **D14** and **D15**) of both SAF and DSS were involved.

Through programming online ER-MS to the constructed two ion transitions (*m*/*z* 507.13 > 211.06 for **M25** and 507.13 > 327.09 for **M23** and **M26**), the OCEs were −38.2 eV and −29.1 eV, respectively. When constructing Q1 > Q3 > Q_LIT_ ion transitions, OCE was defined in q2, and progressive EEs were implemented in LIT chamber to acquire the FEER-MS^3^ spectra. Meanwhile, [M–H]^–^ of each candidate hydrolysis product was permitted to pass q2 cell without dissociation through defining low CE as −10 eV, and thereby acquiring the FEER-MS^2^ spectra of [M–H]^–^ for candidate hydrolysis products.

**Fig. 3:**
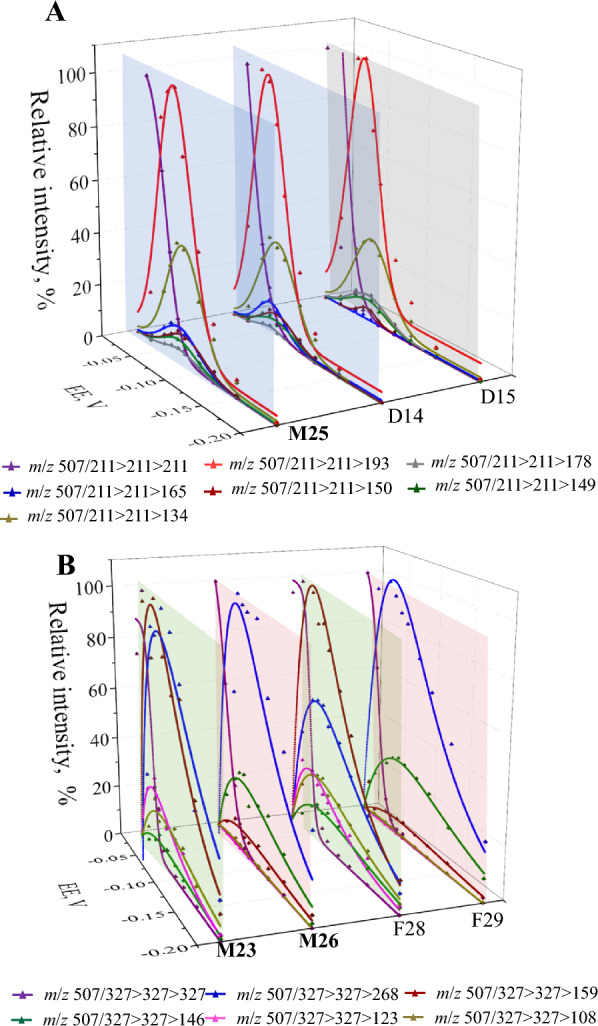
FEER-MS^3^ spectra for monomethylated SAA metabolites compared with FEER-MS^2^ spectra for hydrolysis product anions. (**A**) Overlaid FEER-MS^3^ spectrum of *m*/*z* 211.06 for **M25**, along with FEER-MS^2^ spectra for **D14** and **D15** anions; (**B**) Overlaid FEER-MS^3^ spectra of *m*/*z* 327.09 for **M23** and **M26**, along with FEER-MS^2^ spectra for **F28** and **F29** anions

FEER-MS^3^ spectra of *m*/*z* 507.13 > 211.06 for **M25** together with *m*/*z* 211.06 > 211.06 for **D14** and **D15** are illustrated in Fig. 3A. FEER-MS^3^ spectrum of* y*^−^ (*m*/*z* 211) for **M25** involved the breakdown graphs of *m*/*z* 211 (EE_50_, −0.05626 V), 193 (OEE, −0.06746 V; RII_OEE_, 100%), 178 (−0.07292 V; 3.87%), 165 (−0.07239 V; 12.46%), 150 (−0.07918 V; 10.25%), 149 (−0.07172 V; 7.51%), and 134 (−0.07870 V; 43.54%). The EE_50_ and OEEs of **M25** did not exhibited consistency to **D14** and **D15**, while RII_OEE_ values demonstrated the similarities and the differences. The RII_OEE_ values for each 2^nd^-generation fragment ion of **M25** were consistent with those of **D14** anion (*m*/*z* 193, 100%; *m*/*z* 178, 3.57%; *m*/*z* 165, 13.22%;* m*/*z* 150, 10.08%; *m*/*z* 149, 7.48% and* m*/*z* 134, 38.30%), but did not exhibit such agreement with the **D15** anion (*m*/*z* 193, 100%; *m*/*z* 178, 10.29%; *m*/*z* 165, 1.61%;* m*/*z* 150, 4.77%; *m*/*z* 149, 8.42% and* m*/*z* 134, 32.97%). The detailed information and the RII_OEE_-trend curves of **M25**, **D14**, and **D15** are shown in Additional file 1: Table S3 and Fig. S2. The FEER-MS^3^ spectrum of *y*^−^ for **M25** matched well with the FEER-MS^2^ spectrum for **D14** (3-methyl-DSS) anion. In conclusion, **M25** was configured as 3″-methyl-SAA.

In the same vein, **M23**, **M26**, **F28**, and **F29** were also assayed using post-CID ER-MS strategy to produce FEER-MS^3^ and FEER-MS^2^ spectra (Fig. 3B). Great consistence occurred between FEER-MS^3^ spectrum of *c*^−^ for **M23** and FEER-MS^2^ spectrum for **F28** anion, either of which was made up of the post-CID breakdown graphs of *m*/*z* 327 (EE_50_, −0.04542 V for **M23** and −0.04541 V for **F28**), 268 (OEE and RII_OEE_, −0.06916 V and 91.51% for **M23** *vs*. −0.05896 V and 55.66% for **F28**), 159 (−0.05461 V and 100% for **M23** *vs*. −0.04939 V and 100% for **F28**), 146 (−0.06836 V and 6.75% for **M23** *vs*. −0.06971 V and 12.86% for **F28**), 123 (−0.04614 V and 26.11% for **M23** *vs*. −0.04619 V and 26.12% for **F28**) and 108 (−0.06773 V and 18.58% for **M23** *vs*. −0.06082 V and 25.38% for **F28**). Similarly, there exhibited a significant similarity between FEER-MS^3^ spectrum of *y*^−^ for **M26** and FEER-MS^2^ spectrum for **F29** anion, either of which was composed of breakdown graphs of *m*/*z* 327 (−0.03645 V for **M26** and − 0.03427 V for **F29**), 268 (−0.05664 V and 100% for **M26** *vs*. −0.05594 V and 100% for **F29**), 159 (−0.06762 V and 8.71% for **M26** *vs*. −0.07407 V and 6.00% for **F29**, 146 (−0.06419 V and 29.29% for **M26** *vs*. −0.07071 V and 30.10% for **F29**), 123 (−0.05786 V and 0.08% for **M26** *vs*. −0.07866 V and 0.60% for **F29**) and 108 (−0.04591 V and 1.28% for **M26** *vs*. −0.04017 V and 1.08% for **F29**). The information above is listed in Additional file 1: Table S4 and trend curves of RII_OEE_ are shown in Additional file 1: Fig. S3. The OEE values of **M23**, **M26**, **F28**, and **F29** exhibited no discernible distinction, but EE_50_ value for **M23** closely resembled that of **F28**, while **M26** consistently aligned with the **F29**. Finally, **M23** was tentatively identified as 3'-methyl-SAA and **M26** as either 2-methyl-SAA or 3-methyl-SAA.

Above all, FEER-MS^3^ and FEER-MS^2^ spectra matching is meaningful for structural annotation because RII_OEE_ feature is eligible to capturing diagnostic fragment ions and post CID ER-MS should be a credible strategy to identified fragment ions.

Noteworthily, the post-CID ER-MS strategy could be applicable for identifying any other metabolites, not limited to monomethylated metabolites. In theory, the acquisition of the FEER-MS^3^ spectrum of the concerned 1^st^-generation fragment ion can be achieved by employing the post-CID ER-MS strategy whenever the *c*^–^and *z*^–^ ions generated from ester bond dissociation are detectable in the MS^2^ spectrum. However, glucuronidated and sulfonated substituents are susceptible to neutral loss under CID, and when they are fragmented preferentially over the ester bond, *c*^−^ or *y*^−^ ions are unavailable to acquire FEER-MS^3^ spectra. Subsequent attempts can be made to retain the glucuronosyl and sulfonyl by altering to other dissociation patterns, such as electron capture dissociation (ECD) and electron transfer dissociation (ETD). To some extent, it is extremely challenging to elucidate the exact structures of all metabolites for SAA. Therefore, the current study focused solely on discussing and analyzing methylation as a typical example, which effectively demonstrated the feasibility and practicality of this approach called FEER-MS^3^ and FEER-MS^2^ spectra matching.

### Metabolism channels of SAA in rats

**Fig. 4:**
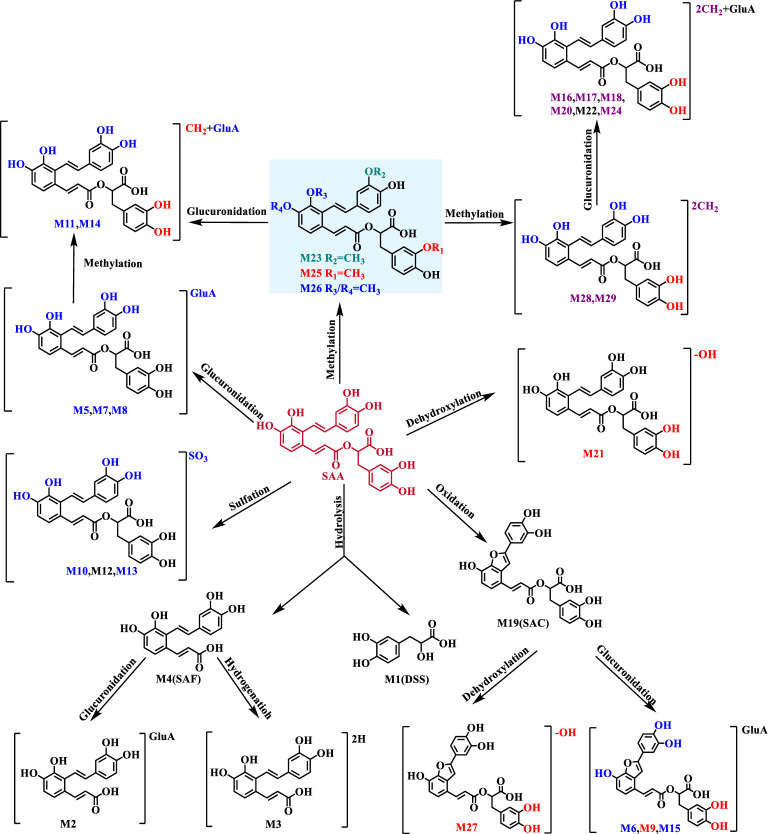
The proposed metabolism channels of salvianolic acid A (SAA) in vivo. Blue: metabolism sites at SAF-unit; Red: metabolism sites at DSS-unit; Purple: metabolism sites at both SAF and DSS, one on each side

After the confidence-enhanced structural annotation for all metabolites (**M1**–**M29**), the metabolic pathways for SAA were proposed (Fig. 4). SAA underwent ester bond hydrolysis and further metabolism to produce **M1**–**M4**. SAA underwent glucuronidation, sulfation, methylation, or a combination of these to generate a series of metabolites (**M5**, **M7**, **M8**, **M10**–**M14**, **M16**–**M18**, **M20**, **M22**–**M26**, **M28**, and **M29**). SAA could directly undergo dihydroxylation to produce **M21**. SAA was able to give birth to SAC (**M19**) *via* oxidation, and subsequently to generate **M27**, **M6**, **M9**, and **M15** through dehydroxylation and glucuronidation. The absence of SAA in plasma indicated that extensive metabolism occurred. All five metabolites detected in plasma were di-methylation plus glucuronidation products. The lower number of metabolites in rat plasma may be attributed to the lower bioconversion activity or the significantly low oral bioavailability and short plasma elimination half-life of SAA in rats [9]. SAC resulting from SAA dehydrogenation and further metabolites were only detected in feces, but not excreted *via* urine, presumably because feces and urine were closely related biliary and renal excretion, respectively.

## Conclusion

Confirmative identification of SAA metabolites in vivo is highly challenging due to the variety of possible metabolism sites. In the present study, one step forward was achieved for structural identification of most metabolites by utilizing the features of ester bond dissociation and the mass differences between featured ions. Post-CID ER-MS strategy was proposed to specify the metabolism sites. And fortunately, the metabolism sites of monomethylated SAA (**M23**, **M25**, and **M26**) were confirmatively determined by matching FEER-MS^3^ spectra of 1^st^-generation fragment ions with FEER-MS^2^ spectra of monomethylated SAF and monomethylated DSS (biosynthetic pioneers). As a result, a total of 29 metabolites of SAA were preliminarily identified in rats. The primary metabolism channels of SAA encompassed hydrolysis, methylation, glucuronidation, sulfation, and oxidation. Overall, this study provides substantial evidence for comprehensively understanding the various metabolic forms of SAA in vivo after oral administration, and the developed strategy should be able to strengthen the identification confidence of complex compounds.

### Supplementary Information

**Additional file 1: Table S1.** Chromatographic and spectrometric information of in vivo metabolites (**F1**–**F30**) for salvianolic acid F (SAF). **Table S2.** Chromatographic and spectrometric information of in vivo metabolites (**D1**–**D15**) for sodium danshensu (sodium DSS). **Table S3.** FEER-MS^3^/MS^2^ spectral information of *m*/*z* 211 for **M25**, **D14**, and **D15**. **Table S4.** FEER-MS^3^/MS^2^ spectral information of *m*/*z* 327 for **M23**, **M26**, **F28**, and **F29**. **Fig. S1.** High-resolution MS/MS spectrum of SAA (A) and the proposed fragmentation pathways (B-1 and B-2). **Fig. S2.** RII_OEE_-trend curves of **M25**, **D14**, and **D15**. **Fig. S3.** RII_OEE_-trend curves of **M23**, **M26**, **F28**, and **F29**.

## Data Availability

All the data used to support the findings of this study are available from the corresponding author upon reasonable request.

## Abbreviations

CE_50_: The collision energy (CE) at 50% survival yield
CID: Collision-induced dissociation
DSS: Danshensu
EE_50_: The exciting energy (EE) at 50% survival yield
EEs: Exciting energies
FCER-MS^2^: Full collision energy ramp-MS^2^
FEER-MS^3^: Full exciting energy ramp-MS^3^
GluA: Glucuronidation
LIT: Linear ion trap
MDs: Molecular descriptors
OCE: Optimal collision energy
OEE: Optimal exciting energy
Online ER-MS: Online energy-resolved mass spectrometry
PITs: Pseudo-ion transitions
Post-CID ER-MS: Post collision-induced dissociation energy-resolved mass spectrometry
RII_max_: Maximum relative ion intensity at OCE
RII_OE__E_: Maximum relative ion intensity at OEE
SAA: Salvianolic acid A
SAF: Salvianolic acid F
