# Supplementary material for: Metabolite identification of salvianolic acid A in rat using post collision-induced dissociation energy-resolved mass spectrometry
Source: Chin Med. 2024 Apr 26;19:64. doi: 10.1186/s13020-024-00931-z (PMC11046765; doi:10.1186/s13020-024-00931-z)
Supplement: Supplementary file 1 — Additional file 1: Table S1. Chromatographic and spectrometric information of in vivo metabolites (F1–F30) for salvianolic acid F (SAF). Table S2. Chromatographic and spectrometric information of in vivo metabolites (D1–D15) for sodium danshensu (sodium DSS). Table S3. FEER-MS3/MS2 spectral information of m/z 211 for M25, D14, and D15. Table S4. FEER-MS3/MS2 spectral information of m/z 327 for M23, M26, F28, and F29. Fig. S1. High-resolution MS/MS spectrum of SAA (A) and the proposed fragmentation pathways (B-1 and B-2). Fig. S2. RIIOEE-trend curves of M25, D14, and D15. Fig. S3. RIIOEE-trend curves of M23, M26, F28, and F29. [file 13020_2024_931_MOESM1_ESM.docx]

**Supplementary Information**

**Metabolite identification of salvianolic acid A in rat using** **post collision-induced dissociation energy-resolved mass spectrometry**

Han Li,^1,2,#^ Ke Zhang,^1,2,#^ Wei Chen,^1,2^ Yuxuan Zhou,^1,2^ Jun Li,^1,2^ Yunfang Zhao,^1,^* Yuelin Song^1,^*

1. Modern Research Center for Traditional Chinese Medicine, Beijing Research Institute of Chinese Medicine, Beijing University of Chinese Medicine, Beijing 102488, China;

2. School of Chinese Materia Medica, Beijing University of Chinese Medicine, Beijing 102488, China.

* Correspondence to:

Dr. Yunfang Zhao, E-mail: yunfang.zhao@163.com;

Prof. Yuelin Song, E-mail: syltwc2005@163.com.

^#^ These two authors contributed equally to this article.

**Figure legends**

**Table S1** Chromatographic and spectrometric information of *in* *vivo* metabolites (**F1**–**F30**) for salvianolic acid F (SAF)

**Table S2** Chromatographic and spectrometric information of *in* *vivo* metabolites (**D1**–**D15**) for sodium danshensu (sodium DSS)

**Table S3** FEER-MS^3^/MS^2^ spectral information of *m*/*z* 211 for **M25**, **D14**, and **D15**

**Table S4** FEER-MS^3^/MS^2^ spectral information of *m*/*z* 327 for **M23**, **M26**, **F28**, and **F29**

**Fig. S1** High-resolution MS/MS spectrum of SAA (A) and the proposed fragmentation pathways (B-1 and B-2)

**Fig. S2** RII_OEE_-trend curves of **M25**, **D14**, and **D15**

**Fig. S****3** RII_OEE_-trend curves of **M23**, **M26**, **F28**, and **F29**

**Table S1** Chromatographic and spectrometric information of *in vivo* metabolites(**F1**–**F30**) for salvianolic acid F (SAF)

| No. | *t*_R_  (min) | Formula | [M−H]^−^ | Error  (ppm) | MS^2^ | Identity | Distribution* |
| --- | --- | --- | --- | --- | --- | --- | --- |
| **SAF** | 16.36 | C_17_H_14_O_6_ | 313.0717 | −0.2 | 269.0830, 159.0456, 109.0302 | Salvianolic acid F(SAF) | U, F |
| **F1** | 10.37 | C_29_H_30_O_18_ | 665.1368 | 1.3 | 489.1077, 313.0744, 269.0847, 175.0264, 159.0459, 109.0302 | SAF diglucuronide | U |
| **F2** | 11.10 | C_29_H_32_O_18_ | 667.1526 | 1.5 | 491.1230, 315.0898, 175.0255 | Hydrogenated SAF di-glucuronide | U |
| **F3** | 11.37 | C_30_H_32_O_18_ | 679.1540 | 3.6 | 503.1236, 327.0904, 283.0991, 175.0262, 159.0468, 123.0461, 108.0223 | Methyl-SAF di-glucuronide | U |
| **F4** | 12.32 | C_23_H_22_O_12_ | 489.1031 | −1.5 | 313.0738, 269.0827, 159.0437, 109.0301 | SAF glucuronide | U |
| **F5** | 12.52 | C_29_H_32_O_18_ | 667.1535 | 2.9 | 491.1235, 315.0903, 175.0262 | Hydrogenated SAF di-glucuronide | U |
| **F6** | 12.79 | C_23_H_24_O_12_ | 491.1196 | 0.2 | 315.0878, 271.0977, 255.0663, 175.0257, 149.0608 | Hydrogenated SAF glucuronide | U |
| **F7** | 13.07 | C_29_H_30_O_18_ | 665.1381 | 3.3 | 489.1069, 445.1164, 313.0733, 269.0840, 175.0261, 159.0469, 109.0305 | SAF di-glucuronide | U |
| **F8** | 13.24 | C_24_H_24_O_15_S | 583.0755 | −1.4 | 407.0423, 327.0869, 309.0793, 283.0994, 268.0775 | Methyl-SAF sulfate and glucuronide | P |
| **F9** | 13.73 | C_24_H_26_O_12_ | 505.1371 | 3.9 | 329.1047, 285.1142, 257.0827, 175.0257 | Methyl-hydrogenated SAF glucuronide | U |
| **F10** | 13.82 | C_23_H_24_O_12_ | 491.1205 | 2.0 | 315.0900, 271.1004, 255.0688, 175.0260, 149.0619 | Hydrogenated SAF glucuronide | U |
| **F11** | 13.99 | C_17_H_16_O_6_ | 315.0877 | 0.9 | 271.0998, 255.0670, 149.0614, 135.0453, 121.0298, 109.0294 | Hydrogenated SAF | F |
| **F12** | 14.21 | C_23_H_22_O_15_S | 569.0623 | 2.9 | 489.1078, 393.0316, 313.0749, 269.0847, 175.0259, 159.0467, 109.0309 | SAF glucuronide and sulfate | P, U |
| **F13** | 14.48 | C_23_H_22_O_12_ | 489.1046 | 1.5 | 313.0742, 269.0844, 159.0468, 109.0309 | SAF glucuronide | P, U |
| **F14** | 14.76 | C_24_H_24_O_15_S | 583.0758 | −0.9 | 503.1185, 407.0443, 327.0888, 159.0456, 123.0461, 108.0225 | Methyl-SAF sulfate and glucuronide | P, U |
| **F15** | 14.88 | C_17_H_14_O_9_S | 393.0285 | −0.2 | 269.0837, 159.0462, 109.0306 | SAF sulfate | U |
| **F16** | 14.96 | C_17_H_16_O_9_S | 395.0438 | −1.1 | 315.0902, 271.1000, 255.0688, 149.0624, 135.0464, 121.0309, 109.0306 | Hydrogenated SAF sulfate | U, F |
| **F17** | 15.21 | C_30_H_32_O_18_ | 679.1517 | 0.2 | 503.1210, 327.0882, 175.0256 | Methyl-SAF di-glucuronide | U |
| **F18** | 15.27 | C_24_H_26_O_12_ | 505.1345 | −1.3 | 329.1040, 285.1152, 175.0255 | Methyl-hydrogenated SAF glucuronide | U |
| **F19** | 15.61 | C_17_H_14_O_9_S | 393.0289 | 0.8 | 269.0842, 159.0465, 109.0308 | SAF sulfate | P, U |
| **F20** | 15.93 | C_24_H_26_O_12_ | 505.1366 | 2.9 | 329.1067, 285.1160, 269.0841, 175.0269 | Methyl-hydrogenated SAF glucuronide | P, U |
| **F21** | 15.94 | C_17_H_16_O_6_ | 315.0879 | 1.5 | 271.0993, 255.0681, 243.0689, 149.0620, 135.0460, 121.0304, 109.0299 | Hydrogenated SAF | F |
| **F22** | 16.27 | C_24_H_24_O_12_ | 503.1213 | 3.6 | 327.0899, 283.0994, 159.0464, 123.0462, 108.0225 | Methyl-SAF glucuronide | P, U |
| **F23** | 17.37 | C_24_H_24_O_12_ | 503.1203 | 1.6 | 459.1322, 327.0901, 309.0728, 283.0984, 268.0763, 175.0249 | Methyl-SAF glucuronide | U |
| **F24** | 17.42 | C_31_H_34_O_18_ | 693.1663 | −1.4 | 517.1363, 341.1038, 175.0233 | Di-methyl-SAF di-glucuronide | P, U |
| **F25** | 18.27 | C_31_H_34_O_18_ | 693.1679 | 1.0 | 517.1361, 341.1045, 326.0816, 175.0254 | Di-methyl-SAF di-glucuronide | P, U |
| **F26** | 18.90 | C_18_H_16_O_9_S | 407.0450 | 1.9 | 327.0891, 312.0656, 283.0999, 268.0763, 159.0460, 109.0299 | Methyl-SAF sulfate | P, U |
| **F27** | 19.13 | C_17_H_14_O_6_ | 313.0733 | 4.9 | 269.0835, 253.0522, 237.0578, 145.0305, 109.0304 | SAF isomer | F |
| **F28** | **19.32** | **C_18_H_16_O_6_** | **327.0882** | **2.4** | **283.0986, 268.0755, 159.0462, 146.0376, 123.0459, 108.0222** | **3′-Methyl-SAF** | **U** |
| **F29** | **20.06** | **C_18_H_16_O_6_** | **327.0869** | −**1.6** | **159.0457, 146.0380** | **2-Methyl-SAF or 3-Methyl-SAF** | **U, F** |
| **F30** | 22.99 | C_19_H_18_O_6_ | 341.1002 | −8.4 | 281.0848, 159.0456, 109.0317 | Dimethyl-SAF | P, U |

*P: plasma; U: urine; F: feces; interested compound (**F28** and **F29**) and relevant information were indicated in bold

**Table S2** Chromatographic and spectrometric information of *in vivo* metabolites(**D1**–**D15**) for sodium danshensu (sodium DSS)

| No. | *t*_R_  (min) | Formula | [M−H]^−^ | Error  (ppm) | MS^2^ | Identity | Distribution* | Reference |
| --- | --- | --- | --- | --- | --- | --- | --- | --- |
| **DSS** | 6.40 | C_9_H_10_O_5_ | 197.0462 | 8.9 | 179.0352, 151.0410, 135.0458, 123.0457, 109.0300 | Danshensu (DSS) | U, F | [1] |
| **D1** | 4.88 | C_15_H_18_O_11_ | 373.0789 | 6.5 | 197.0463, 179.0355, 135.0458 | DSS 4-*O*-glucuronide | U | [2,3] |
| **D2** | 6.10 | C_15_H_18_O_11_ | 373.0789 | 6.4 | 197.0461, 179.0358, 135.0459, 123.0457 | DSS 3-*O*-glucuronide | U | [3] |
| **D3** | 6.17 | C_9_H_10_O_8_S | 277.0025 | −4.6 | 197.0461, 179.0356, 135.0458, 123.0457, 79.9575 | DSS 4-sulfate | P | [1,2] |
| **D4** | 6.44 | C_9_H_10_O_8_S | 277.0027 | −4.0 | 197.0461, 179.0356, 135.0458, 123.0457, 79.9575 | DSS 3-sulfate | U, F | [1,2] |
| **D5** | 6.53 | C_15_H_18_O_11_ | 373.0789 | 6.5 | 197.0462, 179.0357, 175.0257, 135.0458 | DSS 8-*O*-glucuronide | P, U | [-] |
| **D6** | 6.82 | C_9_H_10_O_8_S | 277.0026 | −4.3 | 197.0460, 179.0356, 135.0457, 123.0456, 79.9575 | DSS 8-sulfate | P | [-] |
| **D7** | 7.61 | C_9_H_10_O_7_S | 261.0074 | 4.1 | 181.0513, 163.0408, 119.0508, 79.9575 | Dihydrocaffeic acid 4-sulfate | U | [1,3] |
| **D8** | 7.71 | C_9_H_10_O_7_S | 261.0075 | 4.4 | 181.0514, 163.0408, 135.0458, 119.0508, 79.9576 | Dihydrocaffeic acid 3-sulfate | F | [3] |
| **D9** | 7.78 | C_16_H_20_O_11_ | 387.0942 | 5.2 | 211.0617, 193.0513, 165.0564, 150.0329, 134.0380 | 3-Methyl-DSS 4-*O*-glucuronide | U | [1,2] |
| **D10** | 7.96 | C_10_H_12_O_8_S | 291.018 | −4.8 | 211.0617, 193.0513, 178.0276, 165.0564, 150.0330, 134.0380, 123.0458, 108.0221 | 3-Methyl-DSS 4-sulfate | P, U, F | [1,2] |
| **D11** | 8.46 | C_16_H_20_O_11_ | 387.0942 | 5.3 | 211.0618, 193.0513, 175.0255, 150.0330, 134.0380 | 4-Methyl-DSS 3-*O*-glucuronide | U | [1] |
| **D12** | 8.50 | C_10_H_12_O_8_S | 291.0182 | −4.3 | 211.0618, 193.0513, 150.0330, 134.0380 | 4-Methyl-DSS 3-sulfate | U, F | [1] |
| **D13** | 9.47 | C_9_H_10_O_4_ | 181.0513 | 9.7 | 163.0408, 135.0457, 119.0507, 107.0506 | Dihydrocaffeic acid | P, U, F | [2] |
| **D14** | **9.92** | **C_10_H_12_O_5_** | **211.0618** | **8.1** | **193.0513, 178.0278, 165.0564, 150.0330, 149.0616, 134.0380** | **3-Methyl-dss** | **P, U, F** | **[1-3]** |
| **D15** | **10.67** | **C_10_H_12_O_5_** | **211.0618** | **7.9** | **193.0511, 150.0331, 149.0614; 134.0380** | **4-Methyl-dss** | **P, U, F** | **[1-3]** |

*P: plasma; U: urine; F: feces; interested compound (**D14** and **D15**) and relevant information were indicated in bold


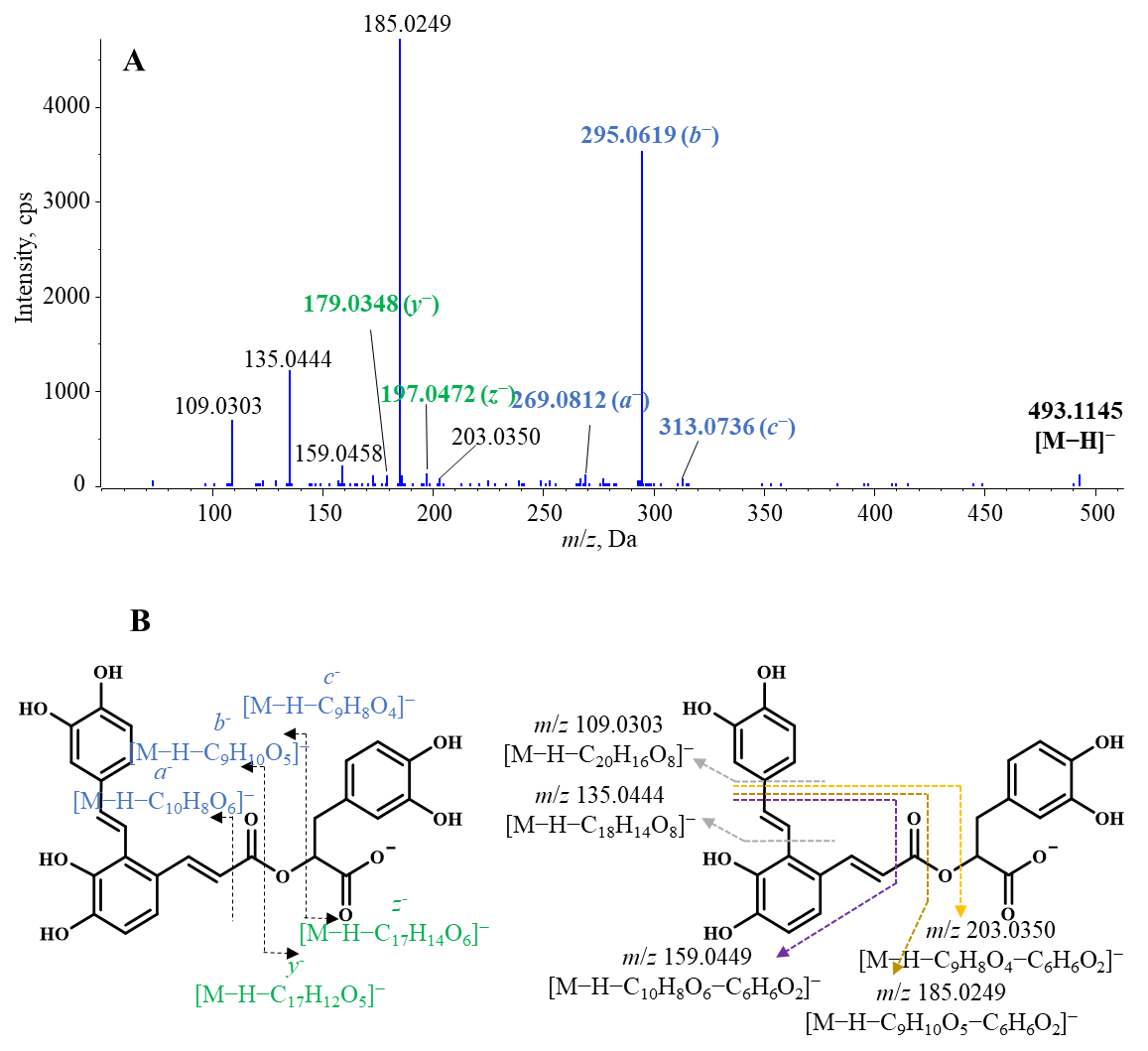


**Fig. S1** High-resolution MS/MS spectrum of SAA (A) and the proposed fragmentation pathways (B)

**Table S3** FEER-MS^3^/MS^2^ spectral information of *m*/*z* 211 for **M25**, **D14**, and **D15**

| Ion transitions | OEE/V, RII_OEE_-**M25** | OEE/V, RII_OEE_-**D14** | OEE/V, RII_OEE_-**D15** |
| --- | --- | --- | --- |
| *m*/*z* 507/211>211>211 | −0.05626 (EE_50_) | −0.04714 (EE_50_) | −0.04517 (EE_50_) |
| *m*/*z* 507/211>211>193 | −0.06746, 100% | −0.05884, 100% | −0.05821, 100% |
| *m*/*z* 507/211>211>178 | −0.07292, 3.87% | −0.06206, 3.57% | −0.06565, 10.29% |
| *m*/*z* 507/211>211>165 | −0.07239, 12.46% | −0.06176, 13.22% | −0.05364, 1.61% |
| *m*/*z* 507/211>211>150 | −0.07918, 10.25% | −0.07020, 10.08% | −0.06472, 4.77% |
| *m*/*z* 507/211>211>149 | −0.07172, 7.51% | −0.06663, 7.48% | −0.06456, 8.42% |
| *m*/*z* 507/211>211>134 | −0.07870, 43.54% | −0.07009, 38.30% | −0.06997, 32.97% |


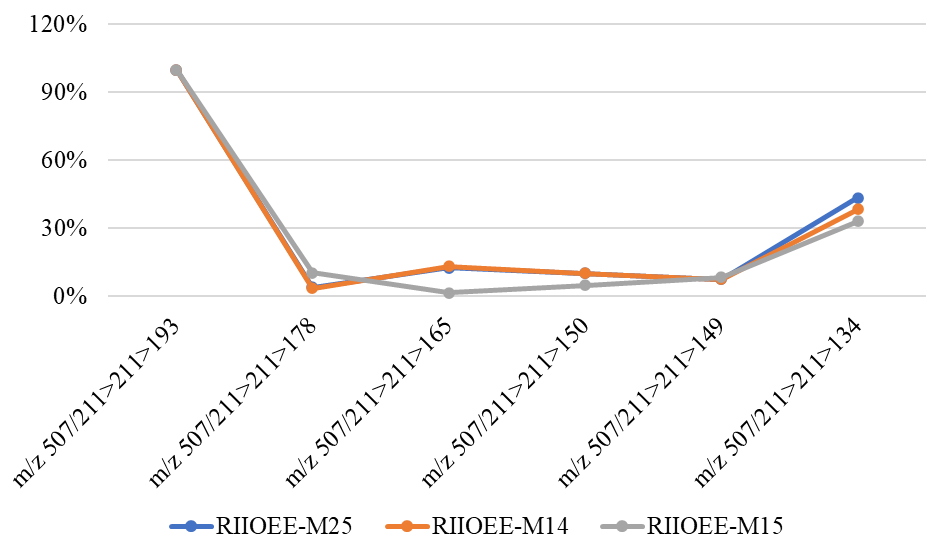


**Fig. S2** RII_OEE_-trend curves of **M25**, **D14**, and **D15**

**Table S4** FEER-MS^3^/MS^2^ spectral information of *m*/*z* 327 for **M23**, **M26**, **F28**, and **F29**

| Ion transitions | OEE/V, RII_OEE_-**M23** | OEE/V, RII_OEE_-**M26** | OEE/V, RII_OEE_-**F28** | OEE/V, RII_OEE_-**F29** |
| --- | --- | --- | --- | --- |
| *m*/*z* 507/327>327>327 | −0.04542 (EE_50_) | −0.03645 (EE_50_) | −0.04541 (EE_50_) | −0.03427 (EE_50_) |
| *m*/*z* 507/327>327>268 | −0.06916, 91.51% | −0.05664, 100% | −0.05896, 55.66% | −0.05594, 100% |
| *m*/*z* 507/327>327>159 | −0.05461, 100% | −0.06762, 8.71% | −0.04939, 100% | −0.07407, 6.00% |
| *m*/*z* 507/327>327>146 | −0.06836, 6.75% | −0.06419, 29.29% | −0.06971, 12.86% | −0.07071, 30.10% |
| *m*/*z* 507/327>327>123 | −0.04614, 26.11% | −0.05786, 0.08% | −0.04619, 26.12% | −0.07866, 0.60% |
| *m*/*z* 507/327>327>108 | −0.06773, 18.58% | −0.04591, 1.28% | −0.06082, 25.38% | −0.04017, 1.08% |


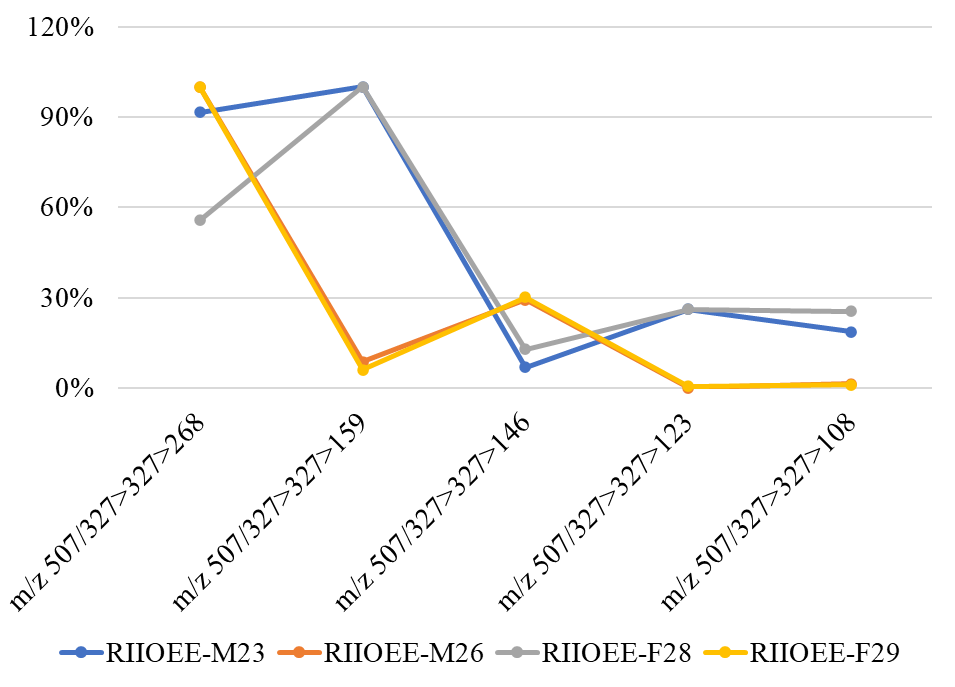


**Fig. S3** RII_OEE_-trend curves of **M23**, **M26**, **F28**, and **F29**

**Reference**

[1] Zhang K, Li H, Shi J, et al. Strategy strengthens structural identification through hyphenating full collision energy ramp-MS^2^ and full exciting energy ramp-MS3 spectra: an application for metabolites identification of rosmarinic acid. Analytica Chimica Acta. 2024; 1296: 342346.

[2] Gu J, Feng L, Zhang M, et al. New metabolite profiles of Danshensu in rats by ultraperformance liquid chromatography/quadrupole-time-of-flight mass spectrometry. J Chromatogr B Analyt Technol Biomed Life Sci. 2014; 955-956: 20-25.

[3] Mei X, Wang Y, Wang Z, et al. Identification of metabolites of Danshensu *in vivo* in rats. Zhongguo Zhong Yao Za Zhi. 2018; 43(19): 3933-3939.
